# Menopausal hormone therapy and risk of sarcoidosis: a population-based nested case–control study in Sweden

**DOI:** 10.1007/s10654-023-01084-3

**Published:** 2024-01-12

**Authors:** Marina Dehara, Susanna Kullberg, Marie Bixo, Michael C. Sachs, Johan Grunewald, Elizabeth V. Arkema

**Affiliations:** 1 Clinical Epidemiology Division, Department of Medicine Solna, Karolinska Institutet, Karolinska University Hospital T2, 171 76 Stockholm, Sweden; 2 Respiratory Medicine Division, Department of Medicine Solna, Karolinska Institutet, Stockholm, Sweden; 3 Center for Molecular Medicine, Karolinska Institutet and Karolinska University Hospital, Stockholm, Sweden; 4 Respiratory Medicine, Theme Inflammation and Ageing, Karolinska University Hospital, Stockholm, Sweden; 5 Department of Clinical Sciences, Obstetrics and Gynecology, Umeå University, Umeå, Sweden; 6 Department of Medical Epidemiology and Biostatistics, Karolinska Institutet, Stockholm, Sweden; 7 Section of Biostatistics, Department of Public Health, University of Copenhagen, Copenhagen, Denmark

**Keywords:** Menopausal hormone therapy, Estrogen, Case–control studies, Risk factors, Sarcoidosis, Women

## Abstract

Sarcoidosis incidence peaks in women between 50 and 60 years old, which coincides with menopause, suggesting that certain sex hormones, mainly estrogen, may play a role in disease development. We investigated whether menopausal hormone therapy (MHT) was associated with sarcoidosis risk in women and whether the risk varied by treatment type. We performed a nested case–control study (2007–2020) including incident sarcoidosis cases from the Swedish National Patient Register (n = 2593) and matched (1:10) to general population controls (n = 20,003) on birth year, county, and living in Sweden at the time of sarcoidosis diagnosis. Dispensations of MHT were obtained from the Swedish Prescribed Drug Register before sarcoidosis diagnosis/matching. Adjusted odds ratios (aOR) of sarcoidosis were estimated using conditional logistic regression. Ever MHT use was associated with a 25% higher risk of sarcoidosis compared with never use (aOR 1.25, 95% CI 1.13–1.38). When MHT type and route of administration were considered together, systemic estrogen was associated with the highest risk of sarcoidosis (aOR 1.51, 95% CI 1.23–1.85), followed by local estrogen (aOR 1.25, 95% CI 1.11–1.42), while systemic estrogen-progestogen combined was associated with the lowest risk compared to never users (aOR 1.12, 95% CI 0.96–1.31). The aOR of sarcoidosis did not differ greatly by duration of MHT use. Our findings suggest that a history of MHT use is associated with increased risk of sarcoidosis, with women receiving estrogen administered systemically having the highest risk.

**Supplementary Information:**

The online version contains supplementary material available at 10.1007/s10654-023-01084-3.

## Introduction

Sarcoidosis is an inflammatory disease of unknown etiology which is characterized by the development of granulomas in any organ, typically in the lungs [1]. Its onset and progression vary; some patients experience acute episodes with spontaneous resolution [2, 3] while others experience a prolonged course resulting in fibrosis and organ function decline [4]. These severe manifestations can result in adverse outcomes including mortality [5], infection [6], heart failure and myocardial infarction [7]. Sarcoidosis can also impact the ability to work, reducing income and quality of life [8]. Treatment primarily involves systemic corticosteroids, typically prescribed for those with debilitating symptoms or signs of disease progression [9].

The incidence of sarcoidosis peaks in women between 50 and 60 years old, estimated to be 15.4 cases per 100,000 per year in Sweden [10]. This age range corresponds to the time of menopause, where there is a significant decrease in estrogen levels. This suggests that certain female hormones, mainly estrogen, may delay sarcoidosis onset in women by improving the aberration of the T-helper (Th)1/Th2 balance in immune response.

The theory that estrogen affects sarcoidosis risk is supported by two previous studies which found that reproductive indicators for endogenous estrogen (produced/synthesized within the body) are protective for sarcoidosis [11, 12]. However, these two studies reported an increased risk associated with exogenous estrogen, in particular menopausal hormone therapy (MHT). MHT is used to mitigate discomfort caused by decreased levels of circulating estrogen and progesterone after menopause [13] and consists of either estrogen alone, or combined estrogen and progestogen (progesterone or synthetic progesterone-like substance called progestin) [14]. The two previous studies on MHT and sarcoidosis, however, were limited due to low power which could have resulted in inadequate statistical precision and reduced ability to detect associations. Moreover, they relied on self-reported MHT use and did not have information on specific types of MHT so the explicit effect of estrogen on sarcoidosis risk was not possible to disentangle. In addition, their results may not be applicable to today since the characteristics of the population receiving MHT and the prescription patterns have changed since 2002 after reports about increased risk of cardiovascular disease and breast cancer [15–18]. Specifically, there has been a shift towards more cautious prescribing practices and a preference for lower doses and shorter durations of treatment. It thus remains unclear whether exposure to MHT is associated with sarcoidosis and whether the risk varies between estrogen alone and combined estrogen-progestogen.

We performed a nested case–control study using information derived from well-established nationwide Swedish population-based registers. Our aim was to investigate whether MHT is associated with risk of developing sarcoidosis in women and whether this risk varies by MHT type, route of administration and duration of use.

## Methods

The data used in this study are part of a larger register linkage which was designed to conduct matched case–control studies to examine risk factors and matched cohort studies to examine outcomes. This register linkage design is an efficient way to conduct multiple investigations of the causes and consequences of sarcoidosis using the same dataset. In this study, we used these data to conduct a case–control study nested within the Swedish population.

### Sarcoidosis cases and general population controls

We identified women with at least two inpatient or outpatient International Classification of Diseases (ICD)-coded visits for sarcoidosis (ICD-8/9 135, ICD-10 D86) in the National Patient Register (NPR; inpatient hospitalizations nationwide since 1987 and visits to outpatient clinics since 2001). A validation study showed that two ICD-coded visits for sarcoidosis in the NPR has a high positive predictive value (0.94) [19]. Women were required to have their first ever visit occurring in 2007–2020, allowing for at least 1.5 years of prescription data before sarcoidosis diagnosis since the Prescribed Drug Register (PDR) became available in July 2005. The PDR captures all prescription dispensations in pharmacies across Sweden since July 2005 including the date and route of administration [20].

Women receiving treatment for sarcoidosis at the time of diagnosis were considered having a more severe sarcoidosis (e.g. debilitating symptoms or organ involvement in need of treatment) [1]. In Sweden, the mainstay of sarcoidosis treatment is systemic corticosteroids and second-line treatment is methotrexate or azathioprine [1]. We classified women as receiving treatment at diagnosis if they had ≥ 1 prescription of either systemic corticosteroids [Anatomic Therapeutic Chemical (ATC) classification codes: H02AB01/02/04/06/07], methotrexate (L01BA01/L04AX03) or azathioprine (L04AX01) in the PDR ± 3 months from their first visit in the NPR listing sarcoidosis. For a subset of cases (n = 108) who were registered in a clinical cohort at Karolinska University Hospital in Stockholm, we retrieved information on sarcoidosis phenotype, i.e. Löfgren or non-Löfgren syndrome.

Women from the general population without sarcoidosis served as controls. Up to 10 population controls without any sarcoidosis visits in the NPR were randomly sampled from the Total Population Register (TPR) and were matched to each unique woman with sarcoidosis on year of birth and residential location at time of sarcoidosis, and required to be living in Sweden at the time the matched case was first identified with sarcoidosis (matching date).

The study population was restricted to women 40 years or older to capture women of menopausal age. Women with a hematologic or lung malignancy diagnosis (ICD-7 162, 163, 200‒205) in the Swedish Cancer Register within 6 months before or after the first visit for sarcoidosis/matching were excluded to avoid including cases where cancer may have been misdiagnosed as sarcoidosis. Additionally, women with a diagnosis of breast cancer, endometrial cancer, cardiovascular disease (stroke, acute myocardial infarction, ischemic heart disease), venous thromboembolism or anticoagulant dispensation before the first visit for sarcoidosis/matching were excluded because they may have a contraindication for MHT use (see flow chart of study population in supplementary Fig. 1 and a list of ICD and ATC codes used for exclusions in supplementary Table 1).

### Exposure: menopausal hormone therapy

To identify history of MHT use, cases and controls were linked to the PDR using each person’s unique identification number. Information on dispensations of MHT [ATC codes: G03C (estrogens), G03D (progestogen, if prescribed in combination with estrogens), G03F (estrogen combined progestogen), G03CX (tibolone; used as an alternative for continuous combined estrogen-progesterone hormone therapy which has androgenic properties); supplementary Table 2] was obtained from the PDR before sarcoidosis diagnosis/matching. Both systemic and local treatments were identified using the pharmaceutical form variable from PDR. Drugs for systemic MHT were defined as oral and transdermal products (i.e. oral tablets, dermal patches and dermal gel) and drugs for local MHT were defined as vaginal products (i.e. vaginal creams, rings and pessaries). A detailed description of the exposure variables is presented in Table1.

**Table 1:** Description of variables used to investigate menopausal hormone therapy (MHT) obtained from the Prescribed Drug Register

Variables	Type	Comment
MHT use	Binary:	
	Never	0 dispensations before diagnosis/matching
	Ever	≥ 1 dispensation of estrogen, estrogen + progestogen or tibolone before diagnosis/matching
Among Ever MHT users		
Type of MHT & route of administration	Categorical:	
	Estrogen (systemic)	≥ 1 dispensation of oral or transdermal estrogen (0 dispensations of estrogen + progestogen, and tibolone, and vaginal estrogen) before diagnosis/matching
	Estrogen + progestogen (systemic)	≥ 1 dispensation oral or transdermal products either as individual estrogen and progestogen components that are co-administered or as combined estrogen-progestogen or as tibolone before diagnosis/matching
	Estrogen (local)	≥ 1 dispensation of vaginal estrogen (0 dispensations of estrogen + progestogen, and tibolone, and oral or transdermal estrogen) before diagnosis/matching
Duration of MHT use	Continuous (3-month increment)	In Sweden, MHT is typically prescribed for 1 year at a time with one dispensation every 3 months. A singular dispensation was assumed to last for 4 months (3 months + 1-month carryover) and we estimated the total duration of MHT by summing up the duration of all individual dispensations; see supplementary methods for how overlaps and gaps in treatment were handled
	Binary: < 12 months; ≥12months	Derived from the duration of use continuous variable
Route of MHT administration	Categorical:	
	Systemic only	≥ 1 dispensation of oral or transdermal products (0 dispensations of vaginal products) before diagnosis/matching
	Local only	≥ 1 dispensation of vaginal products (0 dispensations of oral or transdermal products) before diagnosis/matching
	Systemic + local	≥ 1 dispensation of vaginal products and ≥ 1 dispensation of oral or transdermal products before diagnosis/matching

People with undiagnosed/preclinical sarcoidosis might experience symptoms that mimic menopause, and receive MHT treatment for those symptoms. To mitigate this potential reverse causation, women whose first dispensation occurred within one year before the sarcoidosis diagnosis/matching were not considered exposed in main analyses.

### Other variables

We retrieved demographic information from the TPR including the date of birth, country of birth (Nordic, non-Nordic, missing), and county of residence at diagnosis/matching (classified into healthcare regions: Stockholm, Uppsala-Örebro, West, South, Southeast, and North). From the Longitudinal Integration Database for Health Insurance and Labour Market Studies, we obtained data on education level at time of sarcoidosis diagnosis/matching (≤ 9, 10‒12, ≥ 13 years, missing), gross income in 2005 adjusted to 2019 inflation rate [21] (< 100, 100–< 300, ≥ 300 thousand Swedish krona, missing), and sick leave/disability pension during the year 2005 (0, 1‒49, 50‒199, 200‒364, ≥ 365 days, missing). Sick leave, disability and income were collected from 2005, to assure they were from before exposure and outcome, and not later in time when they could be mediators. When using data on sick leave, disability and income from the year before diagnosis, ORs were within ± 0.02 of the ORs using 2005 data. Women who use MHT may be systematically different in terms of socioeconomic and/or health status than women who do not use MHT. Therefore, we collected information on education and income as proxies for socioeconomic status, and number of days of sick leave/disability pension as a proxy for health status. From the Medical Birth Register, we retrieved data on number of births before sarcoidosis diagnosis/matching. It has been found that number of childbirths is associated with age at menopause [22] and to also be associated with sarcoidosis [11, 12]. A family history of sarcoidosis is the strongest risk factor for sarcoidosis, and is a proxy for genetic risk [23]. We therefore searched for biological first-degree relatives (parents, full siblings and offspring) of cases and controls in the Multi-Generation Register and identified those with at least two sarcoidosis diagnoses in the NPR (family history of sarcoidosis – yes; no) at any point in time.

### Statistical analysis

Characteristics of sarcoidosis cases and general population controls were reported as means with standard deviations, or as proportions. Conditional logistic regression models were used to estimate adjusted odds ratios with 95% confidence intervals (aOR; 95% CI) for the associations between MHT use, type of formulation, route of administration, and duration of use with incident sarcoidosis. MHT type and route of administration were considered together but since some women with systemic estrogen and with combined estrogen-progestogen had a history of local estrogen, we also considered the route of administration alone. We adjusted for age, education, income, sick leave/disability pension, number of births and family history of sarcoidosis.

A subgroup analysis by age at diagnosis/matching (< 60; 60–69; ≥70 years) was conducted to assess whether the association between MHT and sarcoidosis varies by age.

To address the heterogeneity of sarcoidosis, we investigated the association separately for treated and untreated sarcoidosis. Furthermore, to investigate whether misclassification of our register-based definition for sarcoidosis affected our results, we restricted to cases in the Karolinska clinical cohort who have medical record-confirmed diagnoses. Moreover, we examined Löfgren and non-Löfgren syndrome separately using data from the clinical cohort.

We stratified by time from first MHT dispensation to sarcoidosis diagnosis or matching (0 to 7 years) to investigate whether the OR varied by the time since MHT dispensation.

Three separate sensitivity analyses investigating potential misclassification of MHT were conducted. (1) Since the PDR was established in July 2005, we had incomplete information on the first dispensation date (left censoring) so to see if this affected results we included women who had their first ever visit occurring in 2010–2020, allowing for at least 4.5 years of PDR data, (2) we used a stricter definition for MHT, requiring at least two dispensations for MHT in the PDR, and (3) since tibolone has not only estrogenic and progestogenic properties, but also androgenic, we excluded women who received tibolone before sarcoidosis diagnosis/matching.

Since MHT is not used only for menopausal symptoms in a small group of women, we excluded non-menopause indications for MHT to test if those indications affected results (a list of ICD and ATC codes used for exclusions in supplementary Table 3).

We tested the robustness of the results against the potential unmeasured confounding of smoking and obesity using probabilistic bias analysis [24, 25]. Smoking and obesity have been found to be associated with both sarcoidosis [26–29] and women’s sex hormones [30, 31]. The assumptions for the analysis are described in the supplementary methods.

The relative risk (RR) can be computed from the OR as RR = (OR)/[(1 − P) + (P * OR)], where P is the prevalence in the unexposed [10]. With a sarcoidosis prevalence of 160/100,000, an OR of e.g. 1.25 corresponds to RR = 1.20, i.e. practically the same as the OR. We therefore refer to higher/increased odds as higher/increased risk.

Data management and statistical analyses were performed using SAS software (version 9.4; SAS institute Inc., Cary, NC, USA). Forest plots were performed using STATA software (version 16.1).

## Results

We included 2593 newly diagnosed sarcoidosis cases and 20,003 matched general population controls (supplementary Fig. 1). Cases and controls were on average 58 years old (SD ± 11.0), and were comparable with respect to country of birth, education, and number of births (Table 2). Compared to controls, cases were more likely to have income < 100,000 SEK (40.2% vs. 35.0%), have ≥ 365 days on sick leave and disability pension (10.5% vs. 9.0%) and have a family history of sarcoidosis (3.1% vs. 1.1%).

**Table 2:** Characteristics of sarcoidosis cases (N = 2593) and general population controls (N = 20,003), 2007–2020

	Sarcoidosis N = 2593	General population controls N = 20,003
Age at diagnosis/matching, mean (SD)	58.3 (11.2)	58.1 (11.0)
Time from 1st to 2nd diagnosis in months, mean (SD)	5.6 (11.3)	
Time from Prescribed Drug Register start (July 2005) to diagnosis/matching date in years, mean (SD)	5.8 (3.4)	5.6 (3.3)
County of residence at diagnosis/matching, n (%)		
Stockholm	488 (18.8)	3761 (18.8)
Uppsala-Örebro	569 (21.9)	4343 (21.7)
West	480 (18.5)	3684 (18.4)
South	451 (17.4)	3560 (17.8)
Southeast	323 (12.5)	2537 (12.7)
North	282 (10.9)	2118 (10.6)
Country of birth, n (%)^a^		
Nordic	2,230 (86.0)	17,228 (86.1)
Non-Nordic	362 (14.0)	2775 (13.9)
Missing	1 (0.0)	0 (0.0)
Years of education at diagnosis/matching, n (%)		
≤ 9	524 (20.2)	3908 (19.5)
10‒12	1,140 (44.0)	8891 (44.4)
≥ 13	886 (34.2)	6953 (34.8)
Missing	43 (1.6)	251 (1.3)
Income earned in 1000 SEK, n (%)^b^		
< 100	1,041 (40.2)	6998 (35.0)
100‒ < 300	918 (35.4)	7606 (38.0)
≥ 300	538 (20.7)	4752 (23.8)
Missing	96 (3.7)	647 (3.2)
Days on sick leave and disability pension, n (%)^c^		
0	1,766 (68.1)	14,370 (71.8)
1‒49	169 (6.5)	1346 (6.8)
50‒199	201 (7.8)	1365 (6.8)
200‒364	89 (3.4)	466 (2.4)
≥ 365	272 (10.5)	1809 (9.0)
Missing	96 (3.7)	647 (3.2)
Number of births, mean (SD)	1.3 (1.3)	1.4 (1.3)
Family history of sarcoidosis at any point in time, n (%)		
Yes	81 (3.1)	215 (1.1)
No	2512 (96.9)	19,788 (98.9)

^a^Nordic countries include Sweden, Denmark, Norway, Finland, and Iceland

^b^Income earned in year 2005 adjusted for 2019 inflation level. 1.00 SEK ≈ 0.10 USD, 0.09 EUR, or 0.08 GBP

^c^Days on sick leave and disability pension in year 2005. 0 days may include sick leave episodes ≤ 14 days

**Fig. 1:**
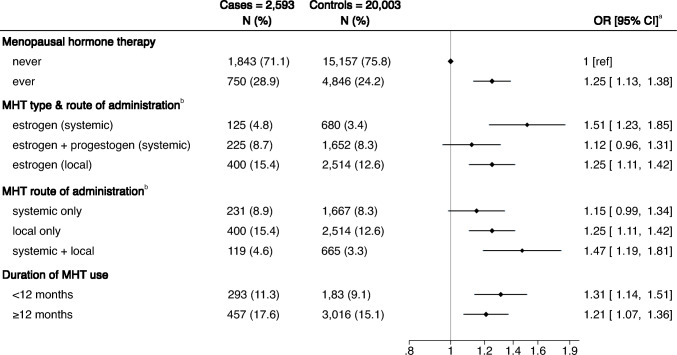
Association between menopausal hormone therapy and sarcoidosis in a nested case–control study in Sweden of 2593 cases and 20,003 controls, 2007–2020. MHT, menopausal hormone therapy; OR, odds ratio; CI, confidence interval. ^a^Odds ratios from conditional logistic regression models adjusted for age, education, income, sick leave/disability pension, number of births and family history of sarcoidosis. ^b^Systemic administration is defined as oral and transdermal products (i.e. oral tablets, dermal patches and dermal gel) and local as vaginal products (i.e. vaginal creams, rings and pessaries)

A larger percentage of cases had a history of MHT use (28.9% vs. 24.2%), and used local estrogen only (15.4% vs. 12.6%) compared to controls (Fig. 1). The average duration of MHT use was 8.1 months (SD ± 19.8) for cases and 6.8 months (SD ± 18.4) for controls.

Compared with never use, ever MHT use was associated with a 25% increased risk of sarcoidosis (aOR 1.25, 95% CI 1.13–1.38; Fig. 1). When type of MHT and route of administration were considered together, systemic estrogen was associated with the highest risk of sarcoidosis (aOR 1.51, 95% CI 1.23–1.85), followed by local estrogen (aOR 1.25, 95% CI 1.11–1.42), and systemic estrogen-progestogen combined (aOR 1.12, 95% CI 0.96–1.31). Women who received both systemic and local treatments had the highest risk of sarcoidosis (aOR 1.47, 95% CI 1.19–1.81), followed by local only (aOR 1.25, 95% CI 1.11–1.42), and systemic only treatment (aOR 1.15, 95% CI 0.99–1.34). The aOR associated with MHT < 12 months, and those exposed ≥ 12 months were 1.31 (95% CI 1.14–1.52) and 1.21 (95% CI 1.07–1.36), respectively (Fig. 1). A 3-month and a 1-year increase in MHT duration was associated with a 1% and a 3% higher risk of sarcoidosis, respectively (aOR 1.01, 95% CI 1.00–1.01 and aOR 1.03, 95% CI 1.00–1.06).

The estimates were similar across age groups (supplementary Table 4). However, the aORs from the main analysis were slightly attenuated for individuals who received sarcoidosis-related treatment around the time of diagnosis (1.14, 95% CI 0.98‒1.33) but not for untreated (1.33, 95% CI 1.16‒1.51; supplementary Table 5). Restricting to cases from the Karolinska clinical cohort yielded a higher OR (aOR 1.56, 95% CI 0.92‒2.63) and this did not differ greatly by Löfgren vs. non-Löfgren syndrome (supplementary Table 6). Stratifying by time from MHT dispensation to sarcoidosis diagnosis or matching, the aOR of sarcoidosis did not materially change (supplementary Table 7‒8). In addition, sensitivity analyses investigating potential misclassification of MHT (among women who had their first ever visit in 2010–2020, defining MHT use as ≥ 2 dispensations, and excluding women who received tibolone; supplementary Table 9) yielded similar results. The estimates did not change considerably after excluding non-menopause indications for MHT (supplementary Table 10). Last, when accounting for unmeasured confounding by smoking, the estimates were slightly higher (OR 1.31, 95% simulation interval 1.27–1.39), and by obesity were almost the same (OR 1.28, 95% simulation interval 1.24–1.39; supplementary Table 11).

## Discussion

In this large nationwide register-based study, we found a 25% increased risk of sarcoidosis associated with a history of MHT use. An increased risk was also observed with different MHT types and route of administration, with women receiving estrogen administered systemically having the highest risk (a 51% increased risk).

Two previous studies found similar results [11, 12]. Specifically, a cohort study using data from the Black Women’s Health Study (BWHS) found that ever MHT use was associated with a 20% increased risk of sarcoidosis [11]. Similarly, a nested case–control study using data from the Northern Sweden Health and Disease Study (NSHDS) showed a 40% higher risk with ever MHT use compared to never use [12]. However, these two studies showed that exposure to endogenous female hormones reduces the risk of sarcoidosis. This indicates that endogenous estrogens may protect against the occurrence of the disease but not exogenous estrogens such as MHT. A similar pattern has been observed with cardiovascular diseases where endogenous estrogen has been found to be protective while this might not be true for estrogen therapy [32]. Recent studies have shown the effect of MHT on cardiovascular disease differs according to timing of initiation, with more beneficial effects when initiated in women < 60 years of age and/or < 10 years after menopause [33] and less favourable effects when initiated in older women and/or > 10 years after menopause [34]. Therefore, the timing of MHT initiation may be an important factor in sarcoidosis too, although we did not find any differences in risk by age group. Future studies should investigate both age at MHT initiation and time since menopause. MHT has been also associated with an increased risk of other immune-mediated inflammatory diseases such as systemic lupus erythematosus [35] and ulcerative colitis [36], indicating that MHT may play a role in the dysregulation of the immune response.

The exact mechanism by which MHT influences sarcoidosis is not known, since there is still an unresolved paradox with respect to the immunomodulating role of estrogens. On one side, estrogens have demonstrated anti-inflammatory activity by inhibiting many pro-inflammatory pathways of innate immunity, adaptive immunity, and inflammatory tissue responses (inhibit the production of Th1 pro-inflammatory cytokines [tumor necrosis factor-α, interferon-γ and interleukin (IL)-2], while they stimulate the production of Th2 anti-inflammatory cytokines [IL-4, IL-10] [37, 38]). On the other side, pro-inflammatory responses have also been shown, including anti-apoptotic effects on immune cells, promotion of neoangiogenesis, and stimulation of B cells [39]. Moreover, estrogens have been shown to activate the mechanistic target of rapamycin (mTOR) and phosphatidylinositol 3-kinase (PI3K) pathways [40, 41], and recent research has suggested that these pathways may play a role in the development and progression of sarcoidosis [42, 43].

The exact mechanism behind the lower risk of sarcoidosis with combined estrogen-progesterone MHT than with only estrogen therapy is not fully understood. However, it is thought that progesterone may play a protective role in reducing the risk of sarcoidosis. Progesterone has anti-inflammatory and immunomodulatory effects [44], which may counteract some of the pro-inflammatory effects of estrogen. Additionally, progesterone has been shown to inhibit the mTOR pathway [45], which has been implicated in the development of sarcoidosis [42, 43].

We observed a 25% increased risk of sarcoidosis associated with local estrogen MHT treatment and we theorized that it could be due to reverse causation. That is, sarcoidosis patients are more likely to receive the diagnosis of urinary tract infections (UTI) which might be partly due to preclinical (asymptomatic) sarcoidosis before diagnosis [46]. Local estrogen administration is more likely to be prescribed to women with a history of UTIs because it reduces the risk of recurrent UTIs [47]. Therefore, we conducted a post-hoc analysis excluding women with a history of UTIs but the OR did not differ greatly from the main analysis (aOR 1.26, 95% CI 1.10–1.43), which does not support our theory. Estrogens administered vaginally can be absorbed into the bloodstream and may have systemic effects [48]. The lower risk associated with local estrogen compared to systemic estrogen (OR 1.25 vs. 1.51, respectively) is consistent with a lower potency of local estrogen administration.

There are several limitations to our study. Information on menopause was not available from nationwide registers, however, we restricted to women 40 years or older to capture women of menopausal age. We did not have information if women received MHT due to menopausal symptoms or other indications not related to menopause. Nevertheless, our estimates remained robust when we excluded non-menopause indications for MHT. There may be some sarcoidosis misclassification, since detailed clinical information in the NPR is not available and sarcoidosis was identified using ICD-coded visits. However, ICD codes for sarcoidosis in the NPR have been shown to have a high positive predictive value [19]. In addition, the odds ratios remained the same (slightly higher) when we restricted to cases in the Karolinska clinical cohort who have medical record-confirmed diagnoses. We only have information on the use of MHT starting in 2005, when data on dispensed medications became available from the PDR. We believe, however, that exposure misclassification is minimal since our estimates remained robust when we included women who had their first ever visit occurring in 2010–2020. Furthermore, when a stricter definition for MHT was used requiring at least two dispensations, our results were similar. There is the possibility of reverse causation bias induced by preclinical sarcoidosis, meaning that symptoms of preclinical sarcoidosis might be mistaken for symptoms of menopause. If this were the case, one would expect that the OR would be higher in the years closest to diagnosis, however, the OR did not vary with time since MHT dispensation (0 to 7 years). Last, we cannot entirely preclude the possibility of unmeasured confounding due to smoking and obesity. However, probabilistic bias analysis showed that these factors have a minimal effect on our results and that we may be underestimating the effect due to the negative confounding of smoking.

A major strength of this study is the use of prospectively collected high-quality population-based data. Using ICD codes for sarcoidosis, which have high validity in the NPR, the study was sufficiently powered to obtain robust inferences. All Swedish residents have universal access to healthcare and we addressed the bias due to socioeconomic and health status by adjusting for education, income and days of sick leave and disability pension. Moreover, after a number of sensitivity analyses examining a range of potential biases, our results did not change. Our study also benefited from detailed information on MHT dispensations from the entire population and considered different types of formulation, and route of administration.

While our study identified a higher risk of sarcoidosis associated with a history of MHT use, the overall decision to initiate MHT should be made on a case-by-case basis. MHT offers significant benefits in alleviating menopause-related hormone deficits, such as relief from vasomotor symptoms, bone density preservation [13], and potentially reduced cardiovascular risks [33]. Moreover, it is essential to understand that the proportion of sarcoidosis cases among women using MHT that can be attributed to MHT is low (attributable proportion 20%). Consequently, the health benefits that MHT can provide to many menopausal women should not be overlooked. However, treatment should be individualized considering various factors, such as a woman’s overall health, medical history, and specific menopausal symptoms.

Interpretations from this study may only be generalizable to older onset sarcoidosis, since sarcoidosis diagnosed at a younger age may differ in terms of etiology (e.g. pathogenetic factors). Lastly, the generalizability of our results may be limited to Northern European ancestry women if the effect of MHT on sarcoidosis risk is different from other ethnic groups. For example, some studies have suggested that estrogen levels are higher in black women and lower in Asian women compared to white women [49–52]. However, given that our results were similar to those reported by the Black Women’s Health Study, we do not think that the external validity of our study is limited.

## Conclusions

Our findings suggest that a history of MHT use is associated with higher risk of sarcoidosis, and women receiving estrogen administered systemically have the highest risk.

### Supplementary Information

Below is the link to the electronic supplementary material.

Supplementary material 1 (DOCX 235 kb)

## References

[1] Grunewald J, Grutters JC, Arkema EV (2019). Sarcoidosis. Nat Rev Dis Primers.

[2] Grunewald J, Eklund A (2009). Löfgren’s syndrome: human leukocyte antigen strongly influences the disease course. Am J Respir Crit Care Med.

[3] Grunewald J, Brynedal B, Darlington P (2010). Different HLA-DRB1 allele distributions in distinct clinical subgroups of sarcoidosis patients. Respir Res.

[4] Statement on sarcoidosis. Am J Respir Crit Care Med. 1999;160:736–55. 10.1164/ajrccm.160.2.ats4-99. 10.1164/ajrccm.160.2.ats4-99 10430755

[5] Rossides M, Kullberg S, Askling J (2018). Sarcoidosis mortality in Sweden: a population-based cohort study. Eur Respir J.

[6] Rossides M, Kullberg S, Eklund A (2020). Risk of first and recurrent serious Infection in sarcoidosis: a Swedish register-based cohort study. Eur Respir J.

[7] Rossides M, Kullberg S, Grunewald J (2022). Risk and predictors of Heart Failure in sarcoidosis in a population-based cohort study from Sweden. Heart.

[8] Arkema EV, Eklund A, Grunewald J (2018). Work ability before and after sarcoidosis diagnosis in Sweden. Respir Med.

[9] Valeyre D, Jeny F, Nunes H (2017). Current medical therapy for sarcoidosis. Semin Respir Crit Care Med.

[10] Arkema EV, Grunewald J, Kullberg S (2016). Sarcoidosis incidence and prevalence: a nationwide register-based assessment in Sweden. Eur Respir J.

[11] Cozier YC, Berman JS, Palmer JR (2012). Reproductive and hormonal factors in relation to incidence of sarcoidosis in US black women: the Black women’s Health Study. Am J Epidemiol.

[12] Dehara M, Sachs MC, Kullberg S (2022). Reproductive and hormonal risk factors for sarcoidosis: a nested case–control study. BMC Pulm Med.

[13] Shuster LT, Rhodes DJ, Gostout BS (2010). Premature menopause or early menopause: long-term health consequences. Maturitas.

[14] Lobo RA (2017). Hormone-replacement therapy: current thinking. Nat Rev Endocrinol.

[15] Buist DS, Newton KM, Miglioretti DL (2004). Hormone therapy prescribing patterns in the United States. Obstet Gynecol.

[16] Hersh AL, Stefanick ML, Stafford RS (2004). National use of postmenopausal hormone therapy: annual trends and response to recent evidence. JAMA.

[17] Hoffmann M, Hammar M, Kjellgren KI (2005). Changes in women’s attitudes towards and use of hormone therapy after HERS and WHI. Maturitas.

[18] Rossouw JE, Anderson GL, Prentice RL (2002). Risks and benefits of estrogen plus progestin in healthy postmenopausal women: principal results from the women’s Health Initiative randomized controlled trial. JAMA.

[19] Ceder S, Rossides M, Kullberg S (2021). Positive predictive value of Sarcoidosis identified in an administrative healthcare registry: a validation study. Epidemiology.

[20] Wettermark B, Hammar N, Fored CM (2007). The new Swedish prescribed Drug Register–opportunities for pharmacoepidemiological research and experience from the first six months. Pharmacoepidemiol Drug Saf.

[21] Statistiska Centralbyrån. Konsumentprisindex (Consumer price index) [Internet]. [cited 2022 May 12]. Available from: www.scb.se/hitta-statistik/statistik-efter-amne/priser-och-konsumtion/konsumentprisindex/konsumentprisindex-kpi/.

[22] Gottschalk MS, Eskild A, Hofvind S (2022). The relation of number of childbirths with age at natural menopause: a population study of 310147 women in Norway. Hum Reprod.

[23] Rossides M, Grunewald J, Eklund A (2018). Familial aggregation and heritability of sarcoidosis: a Swedish nested case–control study. Eur Respir J.

[24] Fox MP, MacLehose RF, Lash TL (2021). Applying quantitative bias analysis to epidemiologic data.

[25] Fox MP, MacLehose RF, Lash TL (2023). SAS and R code for probabilistic quantitative bias analysis for misclassified binary variables and binary unmeasured confounders. Int J Epidemiol.

[26] Carlens C, Hergens MP, Grunewald J (2010). Smoking, use of moist snuff, and risk of chronic inflammatory diseases. Am J Respir Crit Care Med.

[27] Newman LS, Rose CS, Bresnitz EA (2004). A case control etiologic study of sarcoidosis: environmental and occupational risk factors. Am J Respir Crit Care Med.

[28] Ungprasert P, Crowson CS, Matteson EL (2016). Smoking, obesity and risk of sarcoidosis: a population-based nested case–control study. Respir Med.

[29] Valeyre D, Soler P, Clerici C (1988). Smoking and pulmonary sarcoidosis: effect of cigarette smoking on prevalence, clinical manifestations, alveolitis, and evolution of the disease. Thorax.

[30] Segall-Gutierrez P, Du J, Niu C (2012). Effect of subcutaneous depot-medroxyprogesterone acetate (DMPA-SC) on serum androgen markers in normal-weight, obese, and extremely obese women. Contraception.

[31] Pölkki M, Rantala MJ (2009). Smoking affects womens’ sex hormone-regulated body form. Am J Public Health.

[32] Iorga A, Cunningham CM, Moazeni S (2017). The protective role of estrogen and estrogen receptors in cardiovascular disease and the controversial use of estrogen therapy. Biol Sex Differ.

[33] Hodis HN, Mack WJ (2022). Menopausal hormone replacement therapy and reduction of all-cause mortality and cardiovascular disease: it is about time and timing. Cancer J.

[34] Faubion SS, Crandall CJ, Davis L, El Khoudary SR, Hodis HN, Lobo RA, Maki PM, Manson JE, Pinkerton JV, Santoro NF, Shifren JL (2022). The 2022 hormone therapy position statement of The North American Menopause. Soc Menopause.

[35] Rojas-Villarraga A, Torres-Gonzalez JV, Ruiz-Sternberg ÁM (2014). Safety of hormonal replacement therapy and oral contraceptives in systemic Lupus Erythematosus: a systematic review and meta-analysis. PLoS ONE.

[36] Khalili H, Higuchi LM, Ananthakrishnan AN (2012). Hormone therapy increases risk of ulcerative Colitis but not Crohn’s disease. Gastroenterology.

[37] Salem ML (2004). Estrogen, a double-edged sword: modulation of TH1- and TH2-mediated inflammations by differential regulation of TH1/TH2 cytokine production. Curr Drug Targets Inflamm Allergy.

[38] Lorenz TK, Heiman JR, Demas GE (2015). Sexual activity modulates shifts in TH1/TH2 cytokine profile across the menstrual cycle: an observational study. Fertil Steril.

[39] Straub RH (2007). The complex role of estrogens in inflammation. Endocr Rev.

[40] Yu J, Henske EP (2006). Estrogen-induced activation of mammalian target of rapamycin is mediated via tuberin and the small GTPase ras homologue enriched in brain. Cancer Res.

[41] Kazi AA, Molitoris KH, Koos RD (2009). Estrogen rapidly activates the PI3K/AKT pathway and hypoxia-inducible factor 1 and induces vascular endothelial growth factor A expression in luminal epithelial cells of the rat uterus. Biol Reprod.

[42] Pizzini A, Bacher H, Aichner M (2021). High expression of mTOR signaling in granulomatous lesions is not predictive for the clinical course of sarcoidosis. Respir Med.

[43] Zhang B, Dai Q, Jin X (2019). Phosphoinositide 3-kinase/protein kinase B inhibition restores regulatory T cell’s function in pulmonary sarcoidosis. J Cell Physiol.

[44] Fedotcheva TA, Fedotcheva NI, Shimanovsky NL (2022). Progesterone as an anti-inflammatory drug and immunomodulator: new aspects in Hormonal Regulation of the inflammation. Biomolecules.

[45] Lee JH, Lydon JP, Kim CH (2012). Progesterone suppresses the mTOR pathway and promotes generation of induced regulatory T cells with increased stability. Eur J Immunol.

[46] Rossides M, Kullberg S, Askling J (2020). Are infectious Diseases risk factors for sarcoidosis or a result of reverse causation? Findings from a population-based nested case–control study. Eur J Epidemiol.

[47] Rozenberg S, Pastijn A, Gevers R (2004). Estrogen therapy in older patients with recurrent urinary tract infections: a review. Int J Fertil Womens Med.

[48] Krause M, Wheeler TL, Richter HE (2010). Systemic effects of vaginally administered estrogen therapy: a review. Female Pelvic Med Reconstr Surg.

[49] Perry HM, Horowitz M, Morley JE (1996). Aging and bone metabolism in African American and caucasian women. J Clin Endocrinol Metab.

[50] Pinheiro SP, Holmes MD, Pollak MN (2005). Racial differences in premenopausal endogenous hormones. Cancer Epidemiol Biomark Prev.

[51] Haiman CA, Pike MC, Bernstein L (2002). Ethnic differences in ovulatory function in nulliparous women. Br J Cancer.

[52] Shimizu H, Ross RK, Bernstein L (1990). Serum oestrogen levels in postmenopausal women: comparison of American whites and Japanese in Japan. Br J Cancer.

