# Supplementary material for: Menopausal hormone therapy and risk of sarcoidosis: a population-based nested case–control study in Sweden
Source: Eur J Epidemiol. 2024 Jan 12;39(3):313–22. doi: 10.1007/s10654-023-01084-3 (PMC10994872; doi:10.1007/s10654-023-01084-3)
Supplement: Supplementary file 1 — Supplementary material 1 (DOCX 235 kb) [file 10654_2023_1084_MOESM1_ESM.docx]

**Supplement**

**Menopausal hormone therapy and risk of sarcoidosis: a population-based nested case-control study in Sweden**

Marina Dehara, Susanna Kullberg, Marie Bixo, Michael C. Sachs, Johan Grunewald, Elizabeth V. Arkema

**Contents**

SUPPLEMENTARY METHODS 2

Probabilistic bias analysis for unmeasured confounding. 2

Calculation of MHT duration. 4

Attributable proportion. 5

SUPPLEMENTARY TABLES 6

Supplementary Table 1. ICD and ATC codes, contraindicated diseases for MHT 6

Supplementary Table 2. ATC codes for MHT in the PDR 7

Supplementary Table 3. ICD and ATC codes, non-menopause indications in PDR 8

Supplementary Table 4. ORs, age-stratified 9

Supplementary Table 5. ORs, treated and untreated sarcoidosis 10

Supplementary Table 6. ORs, clinical cohort cases, Löfgren, non-Löfgren syndrome 11

Supplementary Table 7. ORs, ≥2 years and no wash-out period 12

Supplementary Table 8. ORs, stratified by recency of dispensation 13

Supplementary Table 9. Sensitivity analyses for MHT misclassification 14

Supplementary Table 10. ORs, excluding non-menopausal indications for MHT 15

Supplementary Table 11. ORs, probabilistic bias analysis 16

SUPPLEMENTARY FIGURES 17

Supplementary Fig. 1. Flowchart of the study population 17

SUPPLEMENTARY METHODS

Creating multiple

imputations, as opposed to single imputations, accounts

for the statistical uncertainty in the imputations. In

addition, the chained equations approach is very ﬂexible

and can handle variables of varying types (e.g. continuous

or binary) as well as complexities such as bounds or

survey skip patterns.

Creating multiple

imputations, as opposed to single imputations, accounts

for the statistical uncertainty in the imputations. In

addition, the chained equations approach is very ﬂexible

and can handle variables of varying types (e.g. continuous

or binary) as well as complexities such as bounds or

survey skip patterns.

Creating multiple

imputations, as opposed to single imputations, accounts

for the statistical uncertainty in the imputations. In

addition, the chained equations approach is very ﬂexible

and can handle variables of varying types (e.g. continuous

or binary) as well as complexities such as bounds or

survey skip patterns.

**Probabilistic bias analysis** **for unmeasured confounding by smoking and obesity**

We used probabilistic bias analysis methods [1, 2] to examine the robustness of our primary estimate for the association between menopausal hormone therapy and sarcoidosis in the presence of unmeasured confounding by smoking and obesity. Data on smoking and obesity are not available in the Swedish nationwide registers used in this study.

We performed 1000 Monte Carlo simulations to check whether the Odds Ratios (OR) from the conventional analysis were robust in the presence of confounding by current smoking and obesity. The assumptions for the probabilistic bias analysis are described as follows:

| Assumptions for the probabilistic sensitivity analysis of the unmeasured confounding effect of smoking and obesity on the association between menopausal hormone therapy and sarcoidosis. | | | | | |
| --- | --- | --- | --- | --- | --- |
|  | Distribution | Estimate | Min | Max | Source |
| Smoking |  |  |  |  |  |
| Prevalence of smoking in women |  |  |  |  |  |
| Exposed | Uniform | 0.23 | 0.22 | 0.24 | [3, 4] |
| Unexposed | Uniform | 0.14 | 0.11 | 0.18 | [5] |
| OR between current smoking and sarcoidosis in women | Uniform | 0.44 | 0.24 | 0.82 | [6] |
| Obesity |  |  |  |  |  |
| Prevalence of obesity in women |  |  |  |  |  |
| Exposed | Uniform | 0.16 | 0.15 | 0.17 | [3, 4] |
| Unexposed | Uniform | 0.20 | 0.16 | 0.25 | [5] |
| OR between obesity and sarcoidosis in women | Uniform | 1.43 | 0.80 | 2.55 | [6] |

The definitions of the bias parameters were informed by prior research [6]. The smoking and obesity prevalences in the exposed group of women were obtained from women aged ≥40 in our previous study using data from the Mammography Screening Project 1995–2006, a population-based prospective cohort [3]. Those data are described in more detail elsewhere [4]. The smoking and obesity prevalences in the unexposed group of women were obtained from Statistics Sweden’s annual surveys of living conditions from women aged 55 to 64 [5].

To estimate a bias-corrected-for-smoking and a bias-corrected-for-obesity OR of sarcoidosis, we repeatedly sampled from the distributions shown in the above table, and calculated the total probability of smoking and obesity (unmeasured confounders) for every individual based on their exposure (menopausal hormone therapy) and outcome (sarcoidosis) status. For every individual, we determined the unknown confounders (binary) by Bernoulli trials based on their probability shown in the above table. We re-estimated the odds ratio from the primary analysis by employing conditional logistic regression, and derived a 95% non-parametric simulation interval accounting for both systematic and random errors from the primary analysis through the utilization of bootstrap techniques. Bootstrap is a resampling method that involves repeatedly resampling the dataset with replacement to create multiple simulated datasets. The confidence intervals were derived using non-parametric approach which means that the interval was constructed based on the simulated distribution of the odds ratio obtained through bootstrap techniques, making it robust and flexible.

**References**

1. Fox MP, MacLehose RF, Lash TL. Applying quantitative bias analysis to epidemiologic data: Springer; 2021.
2. Fox MP, MacLehose RF, Lash TL. SAS and R code for probabilistic quantitative bias analysis for misclassified binary variables and binary unmeasured confounders. Int J Epidemiol 2023 May 4;dyad053. <https://doi.org/10.1093/ije/dyad053>
3. Northern Sweden Health and Disease Study - Mammography Screening Project. <https://www.umu.se/en/biobank-research-unit/provsamlingar-och-register/northern-sweden-health-and-disease-study-vip-monica-and-the-mammography-screening-project/>
4. Dehara M, Sachs MC, Kullberg S, et al. Reproductive and hormonal risk factors for sarcoidosis: a nested case-control study. BMC Pulm Med 2022;22(1):43. <https://doi.org/10.1186/s12890-022-01834-1>
5. Statistiska Centralbyrån. Undersökningar av levnadsförhållanden (ULF/SILC) [Surveys of living conditions]. 2018 [Internet]. [cited 2022 April 26]. Available from: <https://www.scb.se/contentassets/9608d268fa9c40178e30131f03776b76/halsa-2018.xlsx>
6. Dehara M, Sachs MC, Grunewald J, et al. Modifiable lifestyle risk factors for sarcoidosis: a nested case-control study. ERJ Open Res 2023;9(2):00492-2022. <https://doi.org/10.1183/23120541.00492-2022>

**Calculation of menopausal hormone therapy (MHT) duration**

To calculate the total duration of MHT, we first constructed the duration of individual dispensations and then aggregated/summed up the durations.

To calculate the duration of individual dispensation, we assumed that an MHT dispensation typically lasts 3 months and we allow for 1 month of carryover. Thus, a singular dispensation would last 4 months.

| ID | start_date* | end_date calculation | end_date | MHT duration in months |
| --- | --- | --- | --- | --- |
| 200 | 1.1.2006 | 1.1.2006 + 120 days | 1.05.2006 | 4 months |
| 200 | 1.1.2009 | 1.1.2009 + 120 days | 1.05.2009 | 4 months |
| **TOTAL** | | | | **8 months** |
| *****“start_date” is the dispensation date | | | | |

If there was an overlap between two consecutive dispensations (i.e., the interval between the two is less than 4 months ~ 120 days), the duration of the previous dispensation was counted from the date of the dispensation to one day before the next (see below table).

| ID | start_date (dispensation date) | flag_overlap* | next_start_date | end_date calculation | end_date | MHT duration in months |
| --- | --- | --- | --- | --- | --- | --- |
| 201 | 2005-11-04 | 1 | 2006-02-11 | (2006-02-11) –1 day | 2006-02-10 | 3.27 |
| 201 | 2006-02-11 | 1 | 2006-05-18 | (2006-05-18) – 1 day | 2006-05-17 | 3.17 |
| 201 | 2006-05-18 | 1 | 2006-08-01 | (2006-08-01) – 1 day | 2006-07-31 | 2.47 |
| 201 | 2006-08-01 | 1 | 2006-10-05 | (2006-10-05) – 1 day | 2006-10-04 | 2.13 |
| 201 | 2006-10-05 | 1 | 2007-01-29 | (2007-01-29) – 1 day | 2007-01-28 | 3.83 |
| 201 | 2007-01-29 | 1 | 2007-04-18 | (2007-04-18) –1 day | 2007-04-17 | 2.6 |
| 201 | 2007-04-18 | 0 | 2009-03-13 | (2007-04-18) + 120 days | 2007-08-16 | 4 |
| 201 | 2009-03-13 | 1 | 2009-04-22 | (2009-04-22) – 1 day | 2009-04-21 | 1.3 |
| 201 | 2009-04-22 | 0 |  | (2009-04-22) + 120 days | 2009-08-20 | 4 |
| **TOTAL** | | | | | | **26.77 months** |

*flag_overlap=0 means that dispensations do not overlap

*flag_overlap=1 means that dispensations overlap

**Attributable proportion**

The attributable proportion (AP) among the exposed was estimated. AP is a measure used to estimate the proportion of cases of a particular outcome (e.g., a disease) among exposed individuals that can be attributed to the exposure. It is a way to quantify the contribution of an exposure to the occurrence of a specific outcome. So, we estimated the proportion of sarcoidosis cases among women using menopausal hormone therapy (MHT) that can be attributed to MHT.

Calculation of AP:

AP = (Odds Ratio – 1) / Odds Ratio = 1.25 – 1 / 1.25 = 0.2

Thus, 20% of female sarcoidosis cases ≥ 40 years old using MHT could be attributed to MHT.

SUPPLEMENTARY TABLES

| **Supplementary Table 1 \|** International Classification of Disease (ICD) and Anatomical Therapeutic Chemical (ATC) Classification codes used to identify diseases contraindicated for menopausal hormone therapy in the National Patient Register and in the Cancer Register and medication dispensations in the Prescribed Drug Register. | | | | |
| --- | --- | --- | --- | --- |
| **Disease** | **Swedish International Classification of Disease (ICD) codes^a^** | | | **Anatomical Therapeutic Chemical (ATC) Classification codes** |
|  | **10^th^ revision** | **9^th^ revision** | **8^th^ revision** |  |
| Venous thromboembolism^b^ | I80.1–I80.2; I81; I82.2–I82.9; I26 | 415B; 451B; 452; 453C–453D; 453W; 453X | 450–452 | B01AA, B01AB, B01AE, B01AF, B01AX (anticoagulants) |
| Stroke^c^ | I60; I61; I63; I64 | 430; 431; 433–434; 436 | 430; 431; 432–434; 436 |  |
| Ischemic heart disease or acute myocardial  infarction | I20–I25 | 410–414 | 410–414 |  |
|  |  |  | **7^th^ revision** |  |
| Endometrial cancer | C54.1 | 179.9; 182 | 172; 174 |  |
| Breast cancer | C50 | 174 | 170 |  |
| ^a^The Swedish ICD classification system’s 10^th^ revision was in use starting 1997, the 9^th^ revision between 1987 and 1996, the 8^th^ revision between 1969 and 1986, and the 7^th^ revision between 1961 and 1968.  ^b^deep vein thrombosis and pulmonary embolism; ^c^ischaemic stroke, intracerebral haemorrhage, subarachnoid haemorrhage, unspecified stroke | | | | |

| Supplementary Table 2 \| Anatomical Therapeutic Chemical (ATC) codes used to identify menopausal hormone therapy drugs in the Prescribed Drug Register. | | |
| --- | --- | --- |
| Type of therapy | **Formulations/derivatives** | **ATC code** |
| Estrogen only | Estradiol (E2) | G03CA03 |
|  | Estriol (E3) | G03CA04 |
|  | Conjugated estrogens | G03CA57 |
| Tibolone only | Tibolone | G03CX01 |
| Progestogen only | Medroxyprogesterone | G03DA02 |
|  | Dydrogesterone | G03DB01 |
|  | Norethisterone | G03DC02 |
| Estrogen + Progestogen | Norethisterone combined estrogen | G03FA01 |
|  | Medroxyprogesterone combined estrogen | G03FA12 |
|  | Dydrogesterone combined estrogen | G03FA14 |
|  | Drospirenone combined estrogen | G03FA17 |
|  | Norethisterone combined estrogen | G03FB05 |
|  | Medroxyprogesterone combined estrogen | G03FB06 |
|  | Dydrogesterone combined estrogen | G03FB08 |

| **Supplementary Table 3 \|** International Classification of Disease (ICD) and Anatomical Therapeutic Chemical (ATC) Classification codes used to identify non-menopause indications for menopausal hormone therapy in the National Patient Register and in the Prescribed Drug Register. | | | | |
| --- | --- | --- | --- | --- |
| **Disease** | **Swedish International Classification of Disease (ICD) codes^a^** | | | **Anatomical Therapeutic Chemical (ATC) Classification codes** |
|  | **10^th^ revision** | **9^th^ revision** | **8^th^ revision** |  |
| Premature ovarian failure | E28.3; E89.4 | 256.39 | 256.4 |  |
| Endometriosis | N80.0–N80.9 | 617A-G; 617X | 625.30–625.33; 625.38; 625.39 | L02AE (GnRH agonists) |
| Premenstrual dysphoric disorder | F32.81 |  |  |  |
| Menstrual migraine | G43.82; G43.83 | 346.40–346.43 |  |  |
| ^a^The Swedish ICD classification system’s 10^th^ revision was in use starting 1997, the 9^th^ revision between 1987 and 1996, and the 8^th^ revision between 1969 and 1986. | | | | |

| **Supplementary Table 4 \|** Association between menopausal hormone therapy and sarcoidosis in a nested case-control study in Sweden by age at diagnosis/matching, 2007–2020. | | | | | |
| --- | --- | --- | --- | --- | --- |
|  | **OR [95% CI]^a^** | | | | |
|  | **All**  **(cases=2,593, controls=20,003)** | **<60 years**  **(cases=** **1,460, controls=** **11,937)** | | **60-69 years**  **(cases=** **665, controls=** **5,222)** | **≥70 years**  **(cases=** **468, controls=** **2,844)** |
| Menopausal hormone therapy |  |  |  | |  |
| never | ref | ref | ref | | ref |
| ever | 1.25 [1.13–1.38] | 1.21 [1.03–1.41] | 1.29 [1.09–1.53] | | 1.24 [1.00–1.53] |
| Type of MHT & route of MHT administration^b^ |  |  |  | |  |
| estrogen (systemic) | 1.51 [1.23–1.85] | 1.57 [1.16–2.14] | 1.31 [0.91–1.88] | | 1.68 [1.12–2.54] |
| estrogen + progestogen (systemic) | 1.12 [0.96–1.31] | 1.04 [0.84–1.30] | 1.16 [0.89–1.50] | | 1.38 [0.87–2.18] |
| estrogen (local) | 1.25 [1.11–1.42] | 1.25 [1.00–1.57] | 1.36 [1.11–1.66] | | 1.12 [0.88–1.43] |
| Route of MHT administration^b^ |  |  |  | |  |
| systemic only | 1.15 [0.99–1.34] | 1.08 [0.88–1.33] | 1.13 [0.86–1.49] | | 1.48 [0.98–2.22] |
| local only | 1.25 [1.11–1.42] | 1.25 [1.00–1.57] | 1.36 [1.11–1.66] | | 1.12 [0.88–1.43] |
| systemic + local | 1.47 [1.19–1.81] | 1.62 [1.14–2.31] | 1.33 [0.96–1.83] | | 1.62 [1.03–2.54] |
| Duration of MHT use, 3-month increment | 1.01 [1.00–1.01] | 1.01 [0.98–1.01] | 1.01 [1.00–1.02] | | 1.01 [1.00–1.02] |
| Duration of MHT use |  |  |  | |  |
| <12 months | 1.31 [1.14–1.51] | 1.39 [1.15–1.69] | 1.29 [1.00–1.66] | | 1.13 [0.80–1.58] |
| ≥12 months | 1.21 [1.07–1.36] | 1.03 [0.83–1.27] | 1.29 [1.06–1.56] | | 1.29 [1.01–1.63] |
| MHT menopausal hormone therapy; OR odds ratio; CI confidence interval  ^a^Odds ratios from conditional logistic regression models adjusted for age, education, income, sick leave/disability pension, number of births and family history of sarcoidosis.  ^b^Systemic administration is defined as oral and transdermal products (i.e. oral tablets, dermal patches and dermal gel) and local as vaginal products (i.e. vaginal creams, rings and pessaries). | | | | | |

| **Supplementary Table 5 \|** Odds ratios (OR) and 95% confidence intervals (95% CI) of treated and untreated sarcoidosis, separately, in relation to menopausal hormone therapy in a nested case-control study in Sweden, 2007–2020. | | | | |
| --- | --- | --- | --- | --- |
|  | **Treated^a^**  **(cases=1,058, controls=8,158)** | | **Untreated**  **(cases=1,535, controls=11,845)** | |
|  | N cases / controls | OR [95% CI]^b^ | N cases / controls | OR [95% CI]^b^ |
| Menopausal hormone therapy |  |  |  |  |
| never | 758 / 6,151 | ref | 1,085 / 9,006 | ref |
| ever | 300 / 2,007 | 1.14 [0.98–1.33] | 450 / 2,839 | 1.33 [1.16–1.51] |
| Type of MHT & route of MHT administration^c^ |  |  |  |  |
| estrogen (systemic) | 56 / 318 | 1.37 [1.01–1.86] | 69 / 362 | 1.64 [1.25–2.15] |
| estrogen + progestogen (systemic) | 89 / 663 | 1.05 [0.82–1.34] | 136 / 989 | 1.18 [0.96–1.44] |
| estrogen (local) | 155 / 1,026 | 1.13 [0.92–1.38] | 245 / 1,488 | 1.35 [1.14–1.58] |
| Route of MHT administration^c^ |  |  |  |  |
| systemic only | 94 / 704 | 1.06 [0.83–1.34] | 137 / 963 | 1.22 [1.00–1.49] |
| local only | 155 / 1,026 | 1.13 [0.92–1.37] | 245 / 1,488 | 1.35 [1.14–1.58] |
| systemic + local | 51 / 277 | 1.39 [1.01–1.91] | 68 / 388 | 1.53 [1.16–2.01] |
| Duration of MHT use, 3-month increment | 1,058 / 8,158 | 1.00 [0.99–1.01] | 1,535 / 11,845 | 1.01 [1.00–1.02] |
| Duration of MHT use |  |  |  |  |
| <12 months | 117 / 728 | 1.28 [1.03–1.59] | 176 / 1,102 | 1.34 [1.12–1.60] |
| ≥12 months | 183 / 1,279 | 1.06 [0.88–1.28] | 274 / 1,737 | 1.32 [1.13–1.54] |
| MHT menopausal hormone therapy; OR odds ratio; CI confidence interval  ^a^The treated cases are individuals with sarcoidosis receiving pharmacological treatment with systemic corticosteroids, methotrexate, or azathioprine within 3 months of diagnosis.  ^b^Odds ratios from conditional logistic regression models adjusted for age, education, income, sick leave/disability pension, number of births and family history of sarcoidosis.  ^c^Systemic administration is defined as oral and transdermal products (i.e. oral tablets, dermal patches and dermal gel) and local as vaginal products (i.e. vaginal creams, rings and pessaries). | | | | |

| **Supplementary Table 6 \|** Odds ratios (OR) and 95% confidence intervals (95% CI) of sarcoidosis cases retrieved from the Karolinska clinical cohort, and Löfgren syndrome, non-Löfgren syndrome, separately, in relation to menopausal hormone therapy in a nested case-control study in Sweden, 2007–2020. | | |
| --- | --- | --- |
|  | **N cases / controls** | **OR [95% CI]^a^** |
| Sarcoidosis cases from clinical cohort **(cases=108, controls=865)** |  |  |
| Menopausal hormone therapy |  |  |
| never | 79 / 689 | ref |
| ever | 29 / 176 | 1.56 [0.92–2.63] |
| Löfgren’s syndrome  **(cases=26, controls=223)** |  |  |
| Menopausal hormone therapy |  |  |
| never | 21 / 190 | ref |
| ever | 5 / 33 | 1.74 [0.55–5.44] |
| Non-Löfgren’s syndrome  **(cases=82, controls=642)** |  |  |
| Menopausal hormone therapy |  |  |
| never | 58 / 499 | ref |
| ever | 24 / 143 | 1.53 [0.84–2.76] |
| OR odds ratio; CI confidence interval  ^a^Odds ratios from conditional logistic regression models adjusted for age, education, income, sick leave/disability pension, number of births and family history of sarcoidosis. | | |

| **Supplementary Table 7 \|** Odds ratios (OR) and 95% confidence intervals (95% CI) of sarcoidosis associated with menopausal hormone therapy in Sweden, 2007–2020, requiring ≥2 years wash-out period (time from dispensation to sarcoidosis diagnosis or matching date) and an analysis with no wash-out period (0 years from dispensation to sarcoidosis diagnosis or matching). | | | | |
| --- | --- | --- | --- | --- |
|  | **Cases=2,593, Controls=20,003** | | | |
|  | **≥2 years wash-out period** | | **No wash-out period** | |
|  | N cases / controls | OR [95% CI]^a^ | N cases / controls | OR [95% CI]^a^ |
| Menopausal hormone therapy |  |  |  |  |
| never | 1,950 / 15,801 | ref | 1,762 / 14,685 | ref |
| ever | 643 / 4,202 | 1.20 [1.08–1.34] | 831 / 5,318 | 1.29 [1.17–1.42] |
| Type of MHT & route of MHT administration^b^ |  |  |  |  |
| estrogen (systemic) | 107 / 615 | 1.40 [1.13–1.74] | 127 / 732 | 1.46 [1.19–1.78] |
| estrogen + progestogen (systemic) | 194 / 1,445 | 1.08 [0.91–1.27] | 244 / 1,802 | 1.15 [0.99–1.33] |
| estrogen (local) | 342 / 2,142 | 1.22 [1.07–1.40] | 460 / 2,784 | 1.34 [1.18–1.51] |
| Route of MHT administration^b^ |  |  |  |  |
| systemic only | 204 / 1,504 | 1.10 [0.93–1.29] | 230 / 1,744 | 1.12 [0.96–1.30] |
| local only | 342 / 2,142 | 1.22 [1.07–1.40] | 460 / 2,784 | 1.34 [1.18–1.51] |
| systemic + local | 97 / 556 | 1.40 [1.11–1.76] | 141 / 790 | 1.51 [1.24–1.83] |
| Duration of MHT use, 3-month increment | 2,593 / 20,003 | 1.01 [1.00–1.02] | 2,593 / 20,003 | 1.00 [1.00–1.01] |
| Duration of MHT use |  |  |  |  |
| <12 months | 234 / 1,582 | 1.17 [1.01–1.37] | 480 / 3,059 | 1.32 [1.18–1.48] |
| ≥12 months | 409 / 2,620 | 1.22 [1.07–1.38] | 351 / 2,259 | 1.24 [1.08–1.42] |
| MHT menopausal hormone therapy; OR odds ratio; CI confidence interval  ^a^Odds ratios from conditional logistic regression models adjusted for age, education, income, sick leave/disability pension, number of births and family history of sarcoidosis.  ^b^Systemic administration is defined as oral and transdermal products (i.e. oral tablets, dermal patches and dermal gel) and local as vaginal products (i.e. vaginal creams, rings and pessaries).  *≥2-year wash-out:* Only women with ≥2 years (≥730 days) from the first MHT dispensation to sarcoidosis diagnosis /matching were considered as exposed.  *No wash-out period*: Women with ≥0 days from the first MHT dispensation to sarcoidosis diagnosis /matching were considered as exposed. | | | | |

Supplementary Table 8 **|** Odds ratios (OR) and 95% confidence intervals (95% CI) of sarcoidosis associated with at least one menopausal hormone therapy stratified by recency of dispensation to sarcoidosis diagnosis or matching, Sweden 2007–2020.

|  | **Sarcoidosis cases, n (%)**  (N total 2,593) | **General population controls, n (%)**  (N total 20,003) | **OR [95% CI]^a^** |
| --- | --- | --- | --- |
| **Time from first MHT dispensation to sarcoidosis diagnosis /matching** |  |  |  |
| 0 years (≥ 0 days) | 831 (32.1) | 5,318 (26.6) | 1.29 [1.17–1.42] |
| ≥1 year (main analysis) | 750 (28.9) | 4,846 (24.2) | 1.25 [1.13–1.38] |
| ≥2 years | 643 (24.8) | 4,202 (21.0) | 1.20 [1.08–1.34] |
| ≥3 years | 554 (21.4) | 3,546 (17.7) | 1.22 [1.09–1.37] |
| ≥4 years | 474 (18.3) | 2,893 (14.5) | 1.29 [1.14–1.46] |
| ≥5 years | 398 (15.5) | 2,378 (11.9) | 1.30 [1.13–1.48] |
| ≥6 years | 328 (12.7) | 1,920 (9.6) | 1.33 [1.15–1.54] |
| ≥7 years | 268 (10.3) | 1,518 (7.6) | 1.37 [1.17–1.61] |

MHT menopausal hormone therapy; OR odds ratio; CI confidence interval

^a^Odds ratios from conditional logistic regression models adjusted for age, education, income, sick leave/disability pension, number of births and family history of sarcoidosis.

| **Supplementary Table 9 \|** Sensitivity analyses investigating potential misclassification of menopausal hormone therapy (MHT). Association between MHT and sarcoidosis in a nested case-control study in Sweden among women who had their first ever visit for sarcoidosis occurring in 2010–2020 (left), defining MHT use as ≥2 dispensations (middle) and excluding women who received tibolone (right). | | | |
| --- | --- | --- | --- |
|  | **First ever visit for sarcoidosis in 2010–2020**  **(cases=2,120, controls=17,190)** | **≥2 dispensations**  **(cases=2,593, controls=20,003)** | **Excluding tibolone (cases=2,565, controls=19,576)** |
|  | OR [95% CI]^a^ | OR [95% CI]^a^ | OR [95% CI]^a^ |
| Menopausal hormone therapy |  |  |  |
| never | ref | ref | ref |
| ever | 1.24 [1.11–1.38] | 1.18 [1.06–1.31] | 1.25 [1.13–1.38] |
| Type of MHT & route of MHT administration^b^ |  |  |  |
| estrogen (systemic) | 1.48 [1.17–1.86] | 1.35 [1.09–1.68] | 1.50 [1.22–1.84] |
| estrogen + progestogen (systemic) | 1.11 [0.93–1.32] | 1.05 [0.89–1.24] | 1.13 [0.96–1.33] |
| estrogen (local) | 1.26 [1.09–1.45] | 1.22 [1.06–1.40] | 1.24 [1.09–1.41] |
| Route of MHT administration^b^ |  |  |  |
| systemic only | 1.13 [0.95–1.34] | 1.01 [0.85–1.20] | 1.18 [1.01–1.38] |
| local only | 1.26 [1.09–1.45] | 1.22 [1.06–1.40] | 1.24 [1.09–1.41] |
| systemic + local | 1.40 [1.12–1.75] | 1.42 [1.16–1.75] | 1.42 [1.14–1.77] |
| Duration of MHT use, 3-month increment | 1.01 [1.00–1.01] | 1.01 [1.00–1.01] | 1.01 [1.00–1.02] |
| Duration of MHT use |  |  |  |
| <12 months | 1.29 [1.09–1.51] | 1.20 [1.00–1.45] | 1.31 [1.14–1.51] |
| ≥12 months | 1.21 [1.06–1.38] | 1.17 [1.04–1.32] | 1.20 [1.06–1.36] |
| MHT menopausal hormone therapy; OR odds ratio; CI confidence interval  ^a^Odds ratios from conditional logistic regression models adjusted for age, education, income, sick leave/disability pension, number of births and family history of sarcoidosis.  ^b^Systemic administration is defined as oral and transdermal products (i.e. oral tablets, dermal patches and dermal gel) and local as vaginal products (i.e. vaginal creams, rings and pessaries). | | | |

| **Supplementary Table 10 \|** Association between menopausal hormone therapy (MHT) and sarcoidosis in a nested case-control study in Sweden, excluding non-menopausal indications for MHT, 2007–2020. | |
| --- | --- |
|  | **Cases=2,518, Controls=18,944** |
|  | **OR [95% CI]^a^** |
| Menopausal hormone therapy |  |
| never | ref |
| ever | 1.22 [1.11–1.36] |
| Type of MHT & route of MHT administration^b^ |  |
| estrogen (systemic) | 1.40 [1.13–1.74] |
| estrogen + progestogen (systemic) | 1.12 [0.95–1.31] |
| estrogen (local) | 1.25 [1.10–1.42] |
| Route of MHT administration^b^ |  |
| systemic only | 1.11 [0.95–1.30] |
| local only | 1.25 [1.10–1.42] |
| systemic + local | 1.43 [1.15–1.78] |
| Duration of MHT use, 3-month increment | 1.01 [1.00–1.02] |
| Duration of MHT use |  |
| <12 months | 1.28 [1.11–1.48] |
| ≥12 months | 1.19 [1.05–1.34] |
| MHT menopausal hormone therapy; OR odds ratio; CI confidence interval  ^a^Odds ratios from conditional logistic regression models adjusted for age, education, income, sick leave/disability pension, number of births and family history of sarcoidosis.  ^b^Systemic administration is defined as oral and transdermal products (i.e. oral tablets, dermal patches and dermal gel) and local as vaginal products (i.e. vaginal creams, rings and pessaries). | |

| **Supplementary Table 11 \|** Odds ratios and 95% confidence intervals (95% CI) of sarcoidosis associated with menopausal hormone therapy in Sweden, 2007–2020. Probabilistic bias analysis accounting for unmeasured confounding by smoking and obesity. | |
| --- | --- |
|  | Odds ratio (95% CI) |
| **Conventional analysis** |  |
| Random error | 1.25 [1.13–1.38] |
| **Sensitivity analysis^a^** |  |
| Unmeasured confounding by smoking |  |
| Systematic error | 1.31 [1.27–1.39] |
| Systematic and random error | 1.33 [1.19–1.51] |
| Unmeasured confounding by obesity |  |
| Systematic error | 1.28 [1.24–1.39] |
| Systematic and random error | 1.31 [1.16–1.50] |
| Unmeasured confounding by smoking and obesity |  |
| Systematic error | 1.35 [1.27–1.48] |
| Systematic and random error | 1.38 [1.22–1.60] |
| ^a^estimated by simulations based on predetermined assumptions for bias parameters (see supplementary methods). For systematic error analyses, confidence intervals are simulation intervals, and for systematic and random error analyses, confidence intervals are bootstrapped intervals. | |

SUPPLEMENTARY FIGURES


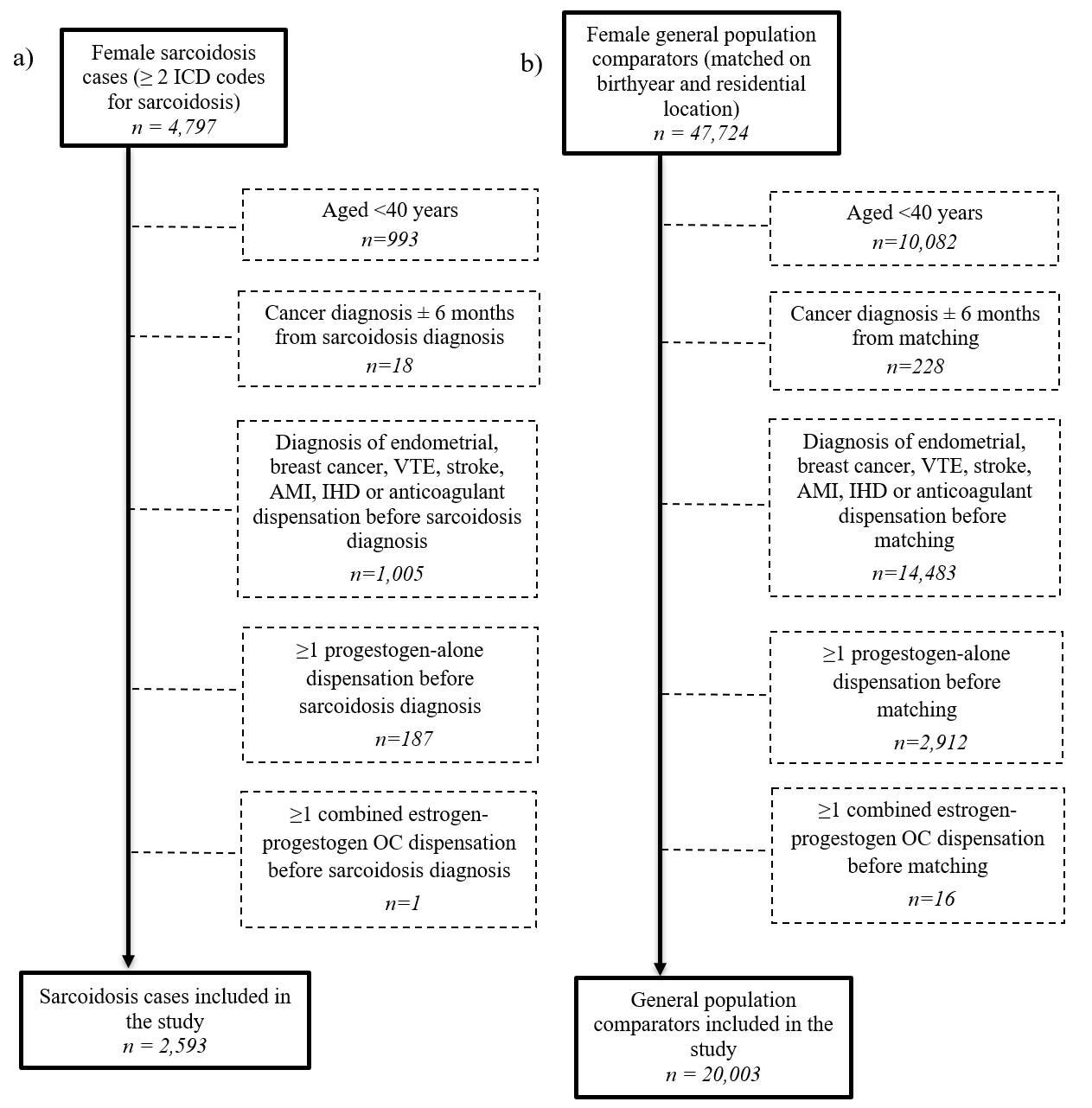


**Supplementary Fig. 1.** Flowchart of the study population comprised of a) female sarcoidosis patients with two or more inpatient or outpatient visits listing a sarcoidosis diagnosis (2007–2020), and b) their matched comparators. ICD: International Classification of Diseases; VTE: Venous thromboembolism; AMI: Acute myocardial infarction; IHD: Ischemic heart disease; OC: Oral contraceptive
